# Three-dimensional topological acoustic crystals with pseudospin-valley coupled saddle surface states

**DOI:** 10.1038/s41467-018-07030-2

**Published:** 2018-11-01

**Authors:** Cheng He, Si-Yuan Yu, Hao Ge, Huaiqiang Wang, Yuan Tian, Haijun Zhang, Xiao-Chen Sun, Y. B. Chen, Jian Zhou, Ming-Hui Lu, Yan-Feng Chen

**Affiliations:** 1 National Laboratory of Solid State Microstructures & Department of Materials Science and Engineering, Nanjing University, Nanjing, 210093 China; 2 Collaborative Innovation Center of Advanced Microstructures, Nanjing University, Nanjing, 210093 China; 3 National Laboratory of Solid State Microstructures & School of Physics, Nanjing University, Nanjing, 210093 China

## Abstract

Topological valley states at the domain wall between two artificial crystals with opposite valley Chern numbers offer a feasible way to realize robust wave transport since only broken spatial symmetry is required. In addition to the valley, spin and crystal dimension are two other important degrees of freedom, particularly in realizing spin-related topological phenomena. Here we experimentally demonstrate that it is possible to construct two-dimensional acoustic topological pseudospin-valley coupled saddle surface states, designed from glide symmetry in a three-dimensional system. By taking advantage of such two-dimensional surface states, a full set of acoustic pseudospins can be realized, exhibiting pseudospin-valley dependent transport. Furthermore, due to the hyperbolic character of the dispersion of saddle surface states, multi-directional anisotropic controllable robust sound transport with little backscattering is observed. Our findings may open research frontiers for acoustic pseudospins and provide a satisfactory platform for exploring unique acoustic topological properties in three-dimensional structures.

## Abstract (web summary)

Valley states can be used to realise topologically protected transport. Here, He et al. show that considering additional degrees of freedom, together with glide symmetry, allow the design of 2D acoustic topological pseudospin-valley coupled saddle surface states in 3D structures.

## Introduction

The discovery of topological phases of matter has renewed our understanding of condensed matter physics over the past few decades^1,2^ and has inspired studies of classical bosonic systems such as photonics^3–12^ and phononics^13–20^. Without considering the difference in spin between fermions (half-integer spin) and bosons (integer spin), their wavefunctions share a similar form associated with a similar topology. This condition gives rise to the search for photonic/phononic analogues of quantum Hall effect with broken time-reversal (TR) symmetry^3,4,14^, topological valley states with a broken mirror or inversion symmetry^10–12,18–20^, Floquet topological states due to temporal (or spatial) modulation^21–23^ and Weyl semimetals with chiral structures^24,25^. However, regarding spin-related topological phenomena, degenerate polarizations or Bloch states must be introduced to construct pseudospins (with pseudo-TR squares to −1)^26^. Therefore, counterparts of the two-dimensional (2D) quantum spin Hall effect^8,15^ and of three-dimensional (3D) topological states^27,28^ for photons/phonons can be designed in principle as electrons in electronic systems by using pseudo-TR instead of natural TR.

For airborne sound such as a spinless wave, an additional degree of freedom (DOF) such as crystal symmetry needs to be considered to construct acoustic pseudospins^29^. Here, we resort to 3D artificial acoustic systems, which provide more flexible platforms to search for this kind of pseudospin among all 230 types of space groups and provide a pre-designable artificial unit structure. Typically, the topological nature is manifested in its character in lower dimension, e.g. 2D systems can possess topological protected one-dimensional (1D) edge or zero-dimensional (0D) corner states^30^, therefore, the 3D models can provide rich topologically protected 2D surface states beyond 1D and 0D lowering from 2D systems. In addition, a typical artificial structure has no more than 10^4^ artificial atoms as a whole, which enable us to accurately manipulate every atom and to deliberately introduce defects, take measurements without limitation of the Fermi level^31^ and to create arbitrary interfaces^32^.

In particular, due to the lack of strong spin–orbit coupling and efficiently TR-breaking method for airborne sound, the valley DOF can provide a convenient way to realize acoustic topological states since only broken mirror (or inversion) spatial symmetry is required. The degenerate point of band structures, such as Dirac degeneracy in 2D case, can be lifted to form the K (K′) valley in the momentum space associated with non-trivial Berry curvature. Although the summation of Berry curvatures over the whole Brillouin zone (BZ) is trivial in TR symmetric cases, robust edge transports still exist along some particular directions.

In this article, we focus on 3D valley acoustic crystals with pseudospin-related topological phenomena. By elaborately designing the symmetries of 3D lattices, the four-fold degenerate point of bulk band structures is lifted to two-fold degenerate valleys, hosting a pair of acoustic pseudospins. The 2D acoustic topological pseudospin-valley coupled saddle surface states and the corresponding anisotropic robust sound transport with little backscattering can be observed in our experiments.

## Results

### Crystal structure and the bulk band structure

**Fig. 1:**
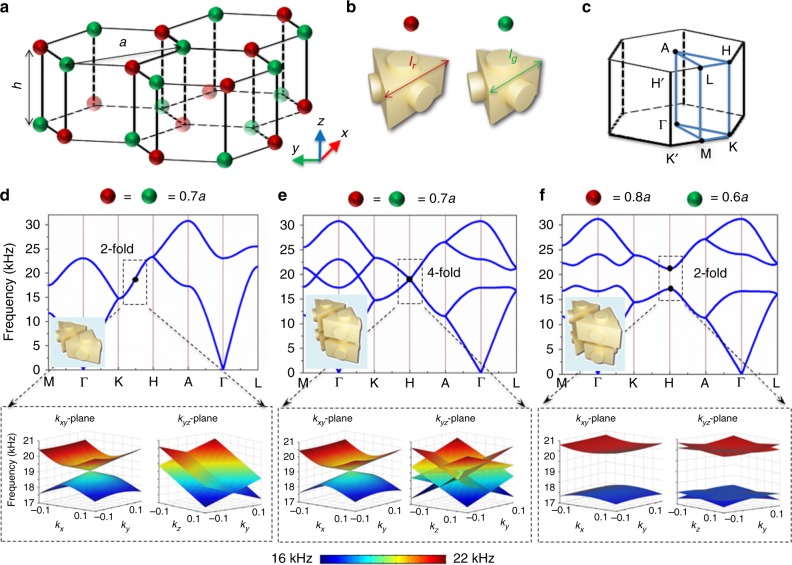
Band structure evolution. **a** Schematic of the crystal structure formed by stacked double-layer honeycomb lattices. Red and green colours represent two types of atoms. **b** The acoustic atom is constructed using a triangular prism cavity with tubes. Different atoms have different side lengths. **c** The schematic for one-half of the first Brillouin zone. **d** The band structures with identical acoustic atoms *l*_*g*_ = *l*_*r*_ = 0.7*a* (one-layer primary unit cell). **e** The case of a double-sized unit cell. **f** The band structures with two different acoustic atoms (*l*_*g*_ = 0.6*a* and *l*_*r*_ = 0.8*a*) possessing glide symmetry. Insets show schematics for the primary unit cells. The lower panels show the bulk bands projected onto *k*_*xy*_ and *k*_*yz*_ planes near the degenerate point (black dots)

Our 3D periodical crystal structure is composed of stacked double-layer honeycomb lattices along the *z* axis, containing two kinds of acoustic atoms in each layer (Fig. 1a). Two adjacent layers show glide symmetry that is the combination of reflection symmetry (*xz* plane) and a translation by half a lattice constant *h* (along the *z* axis). This crystal structure belongs to the non-symmorphic space group No. 194 (P6_3_/mmc). The acoustic atom is a triangular prism cavity with five tubes connecting the nearest neighbours (Fig. 1b). For simplicity, different sidelengths (*l*_*g*_ and *l*_*r*_) of the triangular prisms represent different kinds of acoustic atoms with the other parameters fixed (lattice constant *a* = 8.7 mm, height *h* = 0.54*a*, height of the prism *h*_p_ = *a*/3, radius of the tube *r* = 0.13*a*). One-half of the first BZ is shown in Fig. 1c.

To show the evolution of the band structure, we start from what is likely the simplest 3D structure: a stacked monolayer graphene structure (graphite). The primary unit cell (inset) with all its identical atoms and band structures is shown in Fig. 1d. The side length of the prism is *l*_*g*_ = *l*_*r*_ = 0.7*a*. The bulk band projected onto the *k*_*xy*_ and *k*_*yz*_ momentum planes at the frequency near the centre of the K–H direction is shown in the lower panel. Due to the D_6h_ symmetry, the *k*_*xy*_ projected bulk band is a Dirac cone, where the Dirac point is a two-fold nodal line along the K–H direction. However, just two-fold degenerate states are not enough to construct acoustic pseudospins. The key point is to increase the DOF to form four-fold degeneracy^26^. An efficient method is the BZ folding approach. As shown in Fig. 1e, we choose a double-sized unit cell (associated with one-half of the BZ). Then, the first two bands are folded into four bands with four-fold degenerate states at the H point. The *k*_*xy*_ projected bulk bands become doubly Dirac cones. Due to the unbroken TR symmetry, the states at the H′ point are also four-fold degenerate. Then, we introduce a sublattice structure by choosing two different acoustic atoms (*l*_*g*_ = 0.6*a* and *l*_*r*_ = 0.8*a*) with glide symmetry (Fig. 1f). In this case, a complete band gap is created. More importantly, the four-fold degeneracy is split into two two-fold degeneracies, which can be used to form acoustic pseudospins. The glide symmetry here can be described as ***G***:(*x, y, z*) → (*x,* −*y, z*+*h*). Then, ***G***^**2**^:*(x, y, z*) → *(x, y, z*+2*h)*. Due to the Bloch theorem, the Bloch phase function under lattice translation can be described as $e^{ - i{\boldsymbol{k}} \cdot {\boldsymbol{r}}}$. At the H point (*k*_*z*_ = *π/*2 *h*), ***G*** = $e^{ - i\pi /2}$ (***G***^**2**^ = $e^{ - i\pi }$). The pseudo-TR written as ***GK***, squares to −1 (the complex conjugation ***K*** represents the TR of sound)^26,27^, ensuring that the completely two-fold degenerate Bloch states on the *k*_*z*_ = *π/*2*h* plane form acoustic pseudospins (lower panel of Fig. 1f).

### The pseudospin-valley Chern numbers

Our model is based on a four-band model, which can be treated as a kind of acoustic topological pseudospin-valley states. As shown in Fig. 1e, the four-fold degenerate states at the H (H′) point are doubly Dirac cones projecting onto the *k*_*xy*_ plane. After introducing the sublattice structure (glide), the mirror symmetry respective to the *xz*-plane is broken, with the four-fold degeneracy splitting into two two-fold degenerate acoustic pseudospin± associated with nonzero Berry curvatures. The valley Chern numbers at the H point for the lower two bands (acoustic pseudospin±) (Fig. 1f) are half-integer with opposite signs ${\mathrm{C}}_{\mathrm{H}}^ \pm =\pm 1/2$, while ${\mathrm{C}}_{{\mathrm{H}}^\prime }^ \pm = \mp 1/2$ at the H′ point. Thus, the acoustic pseudospin-valley Chern numbers^33^ can be described as ${\mathrm{C}}_{\mathrm{v}}^{\mathrm{s}} = ({\mathrm{C}}_{\mathrm{H}}^ + - {\mathrm{C}}_{{\mathrm{H}}^\prime }^ + - {\mathrm{C}}_{\mathrm{H}}^ - + {\mathrm{C}}_{{\mathrm{H}}^\prime }^ - )/2$ = +1 (see Supplementary Note 1). It should be noticed that unlike recently realized topological spin-valley-locked states in 2D photonic systems^10^ [where the spins are two intrinsic electromagnetic polarizations with the same signs for the valley Chern numbers at the H(H′) point (associated with ${\mathrm{C}}_{\mathrm{v}}^{\mathrm{s}} = 0$)], our acoustic pseudospins are artificially constructed via crystal symmetry with nontrivial ${\mathrm{C}}_{\mathrm{v}}^{\mathrm{s}}$.

### Acoustic pseudospins for topological saddle surface states

**Fig. 2:**
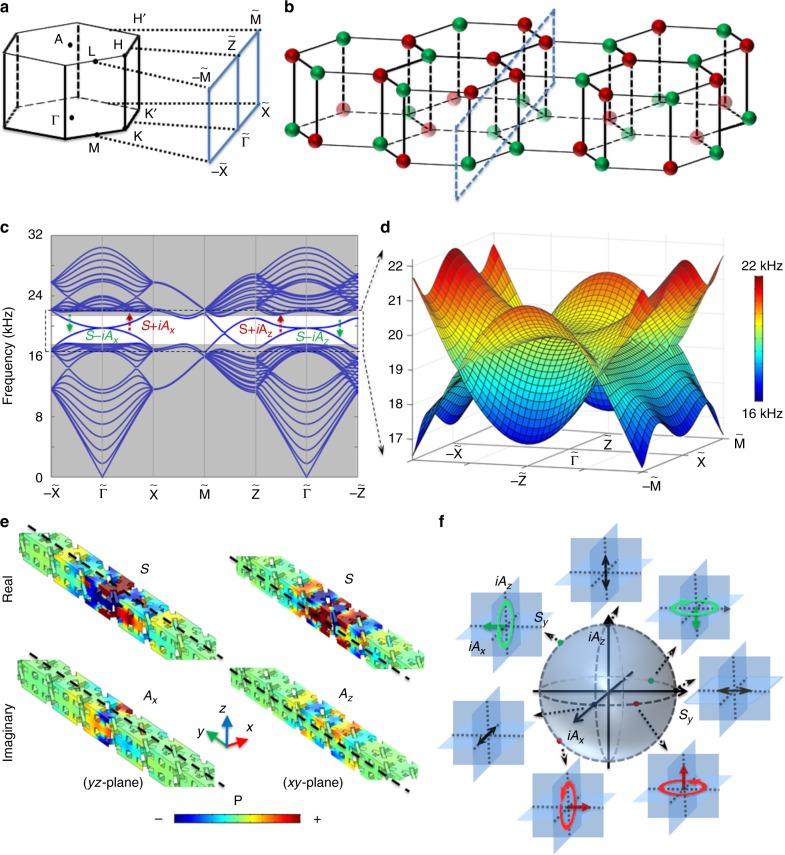
Topological pseudospin-valley states with saddle surfaces. **a** One**-**half of the surface BZ projected onto the *k*_*xz*_ plane. **b** The schematic of the interface. **c** Numerical results for the projected band structures along the high symmetric directions of the surface BZ. The shadow regions indicate the bulk pass bands. **d** Zoom-in surface states for the whole surface BZ. **e** Acoustic pseudospins (marked in **c**) are hybridized by symmetric (*S*) and anti-symmetric (*A*) states with ±*π*/2 phases delay, forming *S* ± *iA*. **f** Acoustic pseudospin sphere on the base of *S* (noted as *S*_*y*_), *iA*_*x*_ and *iA*_*z*_ states, where red and green circles represent opposite chirality, and the corresponding arrows represent pseudospins

To study the 2D topological pseudospin-valley surface states, we introduce a domain wall (zigzag) between two opposite domains on the *xz*-plane. Figure 2a, b shows one-half of the surface BZ projected onto the *k*_*xz*_ plane and a schematic for the interface. Notably, there are two pairs of topological surface states with surface nodal lines, constructed by two opposite saddle surfaces accidentally touching at their saddle points (Fig. 2c, d). Here, we choose four points (marked in Fig. 2c) near the centre of the BZ to investigate the acoustic pseudospins. The acoustic fields are constructed by a symmetric (*S*) real part and anti-symmetric (*A*) imaginary part of the acoustic fields, forming *S* ± *iA* states (Fig. 2e). Then, acoustic pseudospin± can be defined as $\nabla \times$ (*S* ± *iA*). More importantly, the *A* state has two independent components: anti-symmetric respective to the *yz*-plane (noted as *A*_*x*_) and anti-symmetric respective to the *xz*-plane (noted as *A*_*z*_). This result can be attributed to the existence of two mirror symmetric planes perpendicular to the domain wall. Consequently, a full set of acoustic pseudospins on the Bloch sphere (linear, circular or elliptical) can be constructed as shown in Fig. 2f, while in 2D systems, the pseudospins are limited in a 1D space^7,15^. It should be noticed that such a zigzag domain wall has a pseudospin-valley Chern number difference: $\Delta {\mathrm{C}}_{\mathrm{v}}^{\mathrm{s}} = 2$ corresponding to two pairs of topological surface states (Supplementary Figure 3). We can also design a $\Delta {\mathrm{C}}_{\mathrm{v}}^{\mathrm{s}} = 1$ domain wall with only a pair of acoustic pseudospins (see Supplementary Figure 6). It is also worth noting that these acoustic surface states are gapped at the *k*_*xz*_ (armchair) or *k*_*xy*_ interfaces, because the H and H′ points are projected onto the same point of the surface BZ; thus, the valley Chern numbers with opposite signs will be cancelled out (see Supplementary Figure 4).

### Anisotropic robust sound transport

**Fig. 3:**
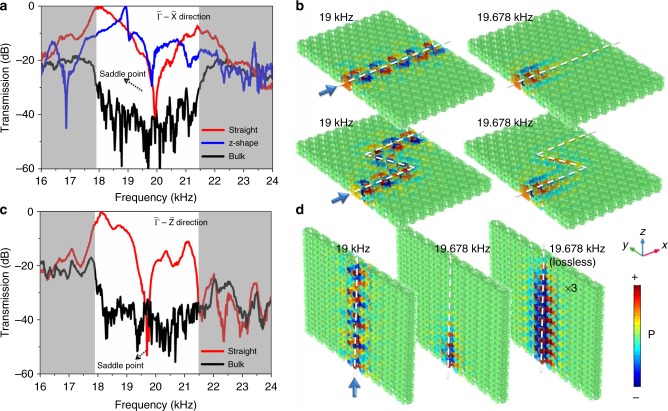
Backscattering suppressed sound transport. **a** Experimental transmission spectra along the ${\tilde{\mathrm \Gamma }}$–${\tilde{\mathrm X}}$ direction, where red, blue and black lines represent the straight, z-shape and bulk conditions, respectively. The shadow regions indicate the bulk pass bands. **b** Simulated field distributions of the acoustic pressure for both straight and z-shape waveguides. **c**, **d** Experimental transmission spectra along the ${\tilde{\mathrm \Gamma }}$–${\tilde{\mathrm Z}}$ direction and corresponding field simulations. The loss is considered in simulations, except for one case (noted as lossless) for comparison. The white dashed lines indicate the interfaces

In our experiment, we choose two orthogonal directions to show multi-directional robust acoustic pseudospin-valley transport (Supplementary Figure 1). Figure 3a shows the measured transmission spectra (10 periods) for surface states along the ${\tilde{\mathrm \Gamma }}$–${\tilde{\mathrm X}}$ direction with both straight (red line) and z-shape (blue line) waveguides. In contrast, the measured transmission spectra for bulk states are shown by the black line, with a relative band gap width of over 15%. Following the incidence of the pseudospin+ acoustic wave, the transmissions maintain a very high transmission value in the bulk band gap frequency region for both straight and z-shape waveguides, indicating a strongly suppressed backscattering property (Supplementary Figure 2). The overall transmission of the saddle surface states is 20 dB larger than that of the bulk within the bulk band gap, representing the surface’s gapless behaviour, except for a transmission dip near 19.678 kHz according to the quadratic saddle point. Near the saddle point, the extremely flat dispersion results in the rapidly enhanced state intensity of sound; thus, the transmission is intensely attenuated even at a very low loss. The simulation results for the pressure field at frequencies of 19 kHz (in bulk band gap) and 19.678 kHz (near the saddle point) are shown in Fig. 3b. In simulations, the loss is introduced by adding an imaginary part (10^−3^) for the sound speed. On the other hand, even without the nonlinear effect of loss, the transmission near the saddle point is still sharply decreased because of the diminishing group velocity down to zero. A similar robust transmission along the ${\tilde{\mathrm \Gamma }}$–${\tilde{\mathrm Z}}$ direction is experimentally observed (Fig. 3c), matching well with the simulation results (Fig. 3d). The lossless condition at the saddle point is also provided for comparison (right panel).

**Fig. 4:**
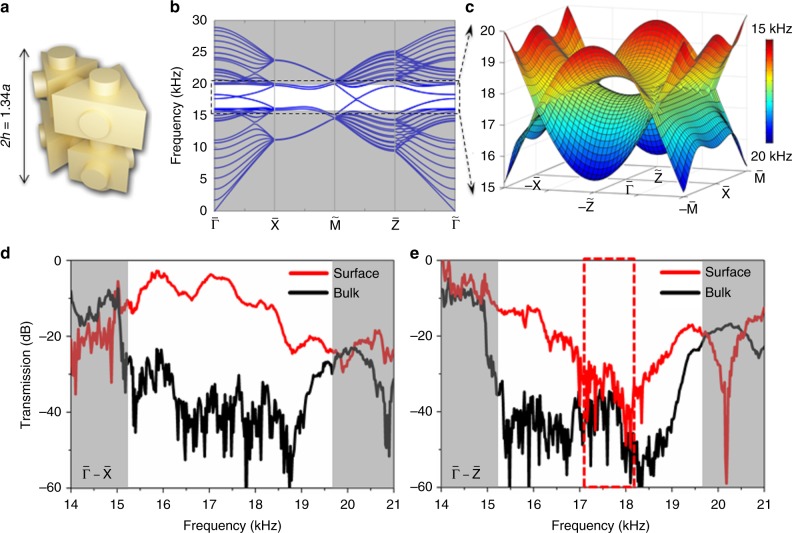
Hyperbolic behaviour for the acoustic topological pseudospin-valley states. **a** The unit cell with increased height. **b** Numerical results for the projected band structures along the high symmetric directions of the surface BZ. **c** Zoom-in 3D view of the surface states in an eye shape. **d**, **e** Experimental transmission spectra along the ${\tilde{\mathrm \Gamma }}$–${\tilde{\mathrm X}}$ and ${\tilde{\mathrm \Gamma }}$–${\tilde{\mathrm Z}}$ directions, respectively. Dashed box represents the frequency region of the eye. The shadow regions indicate the bulk pass bands

To further verify such saddle surface states, we increase the height (*h*) between two layers from 0.54*a* to 0.67*a* (Fig. 4a). The interaction between the two layers is weaker. Thus, the whole band structures show a slight redshift. Interestingly, two opposite saddles are separated to form an eye-shape (Fig. 4b, c). The experimental transmission spectra along the ${\tilde{\mathrm \Gamma }}$–${\tilde{\mathrm X}}$ and ${\tilde{\mathrm \Gamma }}$–${\tilde{\mathrm Z}}$ directions are shown in Fig. 4d, e. There is no obviously decreased transmission along the ${\tilde{\mathrm \Gamma }}$–${\tilde{\mathrm X}}$ direction. However, due to the eye opening, a wider transmission dip along the ${\tilde{\mathrm \Gamma }}$–${\tilde{\mathrm Z}}$ direction can be found than that in Fig. 3c. The dashed box represents the frequency region of the eye. Based on such strong hyperbolic behaviour for the topological saddle surface states, multi-directional anisotropic controllable robust sound transport with little backscattering can be obtained (see Supplementary Figures 7–10).

## Discussion

In summary, we experimentally demonstrate 2D acoustic pseudospin-valley coupled saddle surface states in 3D topological acoustic crystals generated due to glide-symmetry design^29^. Compared to the electronic topological crystalline insulator with saddle dispersion in condensed matter physics^34,35^, this acoustic model exhibits surface nodal lines for the surface states, which can hardly shrink to a Dirac cone because of the lack of intrinsic acoustic spins (Supplementary Figure 5). Our acoustic pseudospins (satisfying pseudo-TR) are constructed by crystal symmetry which cannot be kept intact on the domain wall. Unlike the intrinsic spins of electrons, these acoustic pseudospins are gradually changed along the surface, e.g. becoming linear pseudospins at the saddle point. However, this 3D acoustic topological model still shows strongly suppressed backscattering behaviour on the whole 2D surface, which resembles the 2D topological cases with a tiny gap in the middle of the 1D edge states^7,15,36^ due to spatial symmetry breaking on the boundary. The results we revealed here may pave the way towards acoustic pseudospins and valleytronics in 3D structures^37^. The saddle surface states could be applied to realize hyperbolic pseudospin filters^37^. The robust pseudospin-valley propagation within a large topological band gap and the extremely flat dispersion near the saddle point may give rise to an ultraslow sound, ultrahigh-*Q* acoustic resonator.

## Methods

### Experiments

Our samples are fabricated by 3D printing with commercial low-viscosity liquid photopolymer materials (Somos Imagine 8000). The tolerance of the fabrication is ±0.1 mm, which is less than 5% compared to the smallest feature size of 2.2 mm in our model. Due to the fabrication tolerance of different samples, the measured transmission spectra for the saddle points in Fig. 3a, c show a slight blue or redshift. A B&K-4939-2670 microphone acts as a detector, which is placed 1 cm from the boundary with its response acquired and analysed in B&K-3560-C. The frequencies are swept from 14 to 24 kHz with an increment of 0.02 kHz. The experimental transmission spectra plotted in this article are normalized to the acoustic wave transmission through the same distance in air. The slight deviation recorded in experiments is due primarily to the frequency dependent coupling into and out efficiency.

### Simulations

Numerical investigations used to calculate band structures (Figs. 1d–f, 2c, d and 4b) and field distributions (Figs. 2e and 3b, d) are conducted by using an acoustic model in commercial FEM software (COMSOL MULTIPHYSICS). Due to the large acoustic impedance mismatch between air and photopolymer material (modulus 2765 MPa, density 1.3 g cm^−3^), the models in the numerical calculation are constructed using only acoustic cavities with hard boundaries, without considering the polymer background. The density and velocity of sound are chosen to be 1.25 kg m^−3^ and 343 m s^−1^, respectively. The numerical results agree well with experimental results.

## Electronic supplementary material

Supplementary Information

## Data Availability

The data that support the plots within this paper and other findings of this study are available from the corresponding author upon request.
