# Supplementary material for: Three-dimensional topological acoustic crystals with pseudospin-valley coupled saddle surface states
Source: Nat Commun. 2018 Nov 1;9:4555. doi: 10.1038/s41467-018-07030-2 (PMC6212403; doi:10.1038/s41467-018-07030-2)
Supplement: Supplementary file 1 — Supplementary Information [file 41467_2018_7030_MOESM1_ESM.pdf]

Supplementary information for

Three-dimensional topological acoustic crystals with pseudospin-valley coupled saddle surface states

He et al.

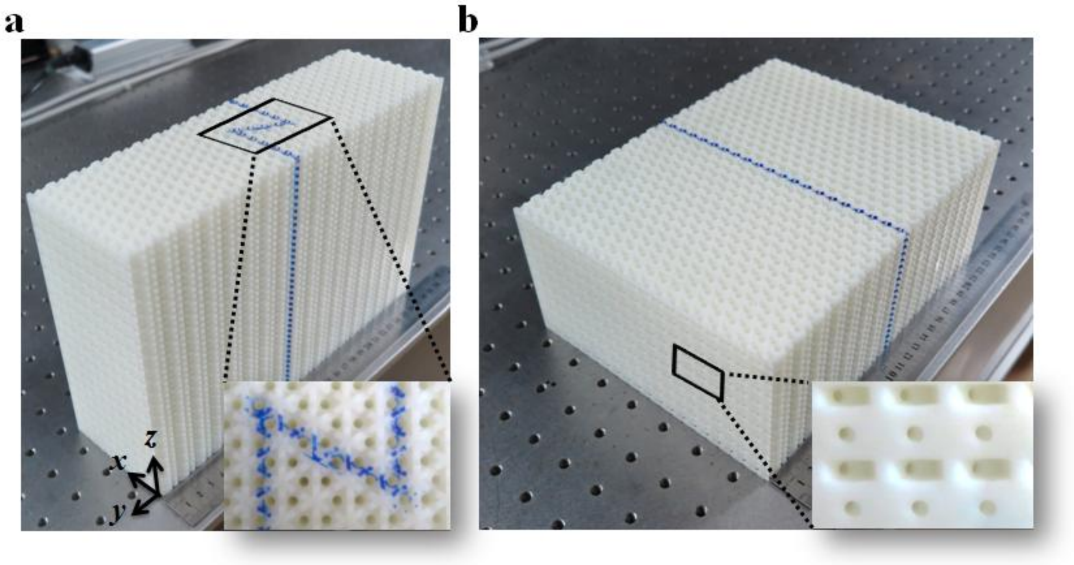

Supplementary Figure 1 | 3D acoustic samples. a, Sample with the z-shape interface used to check the robustness. The experiment transmission spectrum corresponds to the blue line in Fig. 3a of main text. Inset shows the top view of zoom-in photo. **b,** The sample used to measure transmission spectrum along *z* axis, corresponding to Fig. 4e of main text. Inset shows the lateral view of zoom-in photo. Blue lines marked on the samples represent the interfaces.

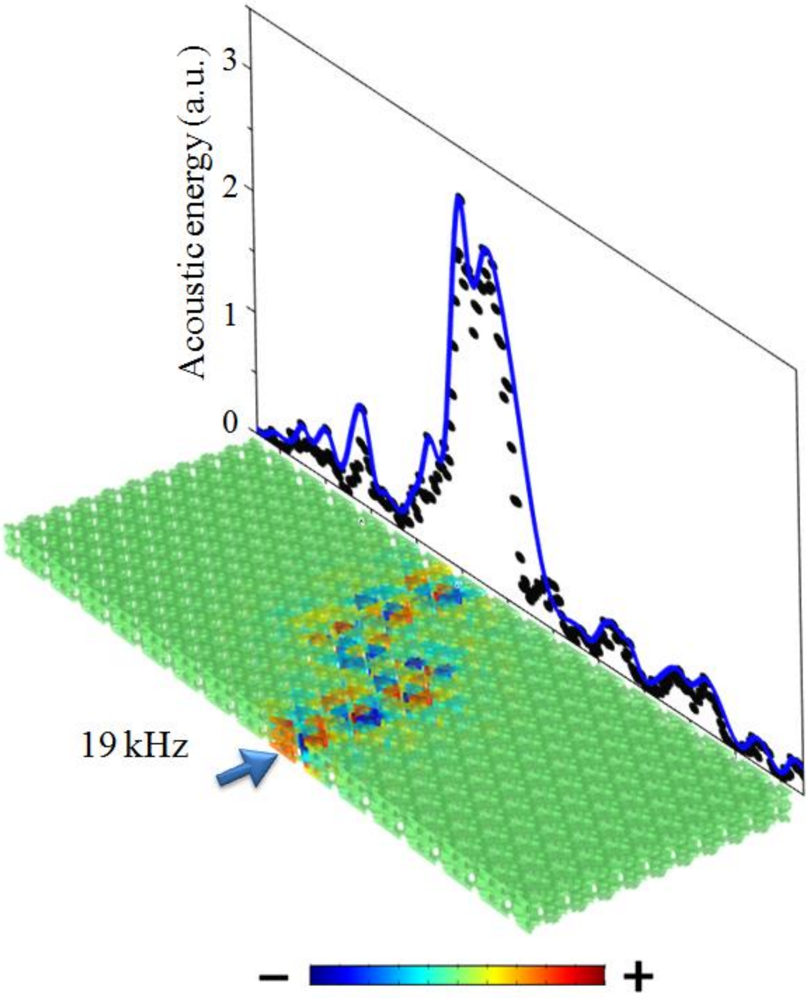

Supplementary Figure 2 | Profile of acoustic energy at output port. To verify the acoustic wave propagating along the z-shape waveguide, we measure the acoustic energy along the output port of Supplementary Figure 1a (black dots). The blue line is the upper envelope of experiment data, which shows that the acoustic wave is well localized in the waveguide. Operation frequency is chosen to be 19 kHz. Color picture represents the simulation result of acoustic pressure (bottom panel).

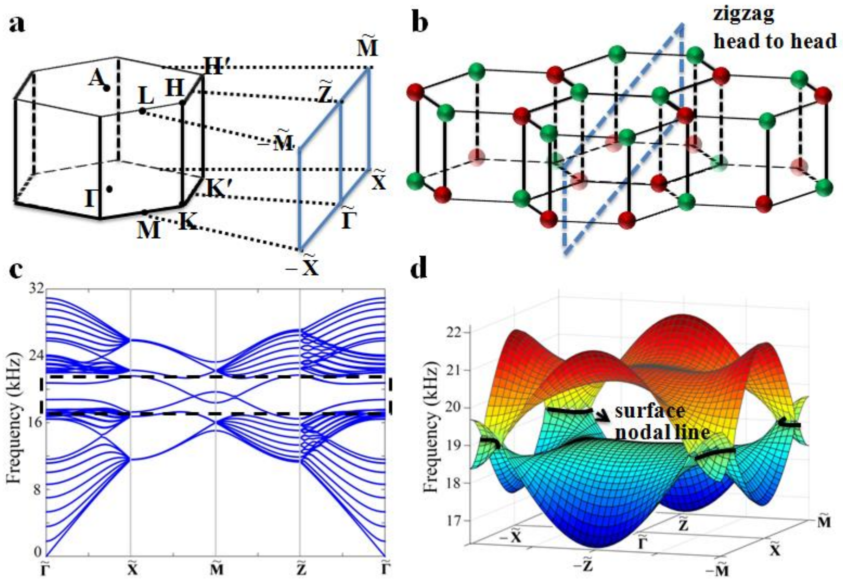

Supplementary Figure 3 | Acoustic topological pseudospin-valley states projected onto another type of zigzag domain wall. a, One-half of the surface Brillouin zone projected onto the k_{xz} plane. **b,** The schematic for the head to head zigzag interface. **c,** Numerical results for the projected band structures along the high symmetric directions of the surface BZ. **d,** Zoom-in surface states in the whole surface BZ. The surface nodal rings are around the corner of surface BZ.

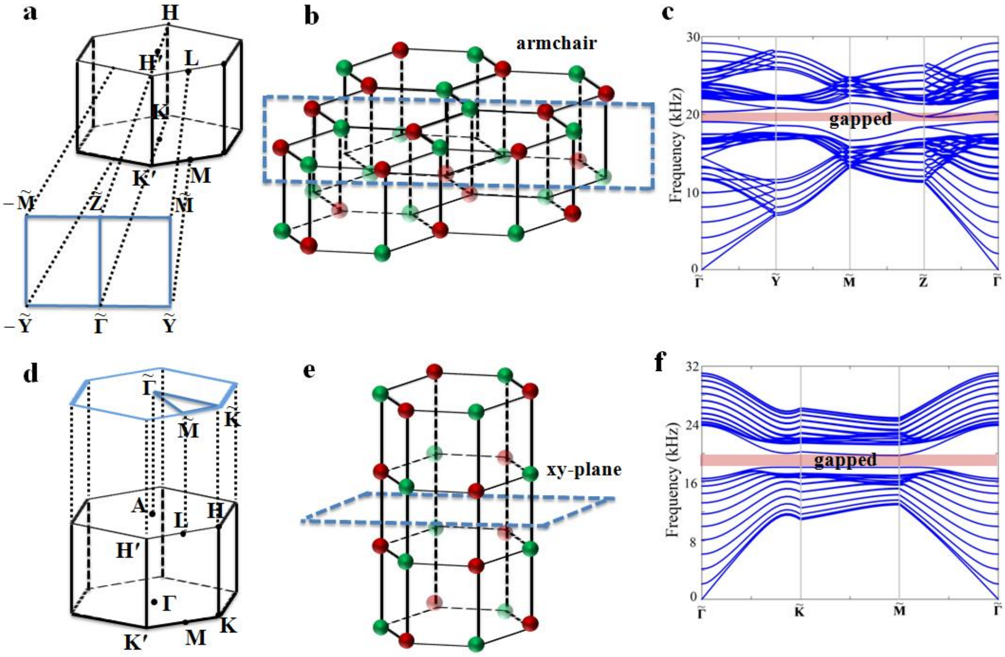

Supplementary Figure 4 | Surface states projected onto the k_{yz} and k_{xy} planes. a, One-half of the surface BZ projected onto the k_{yz} plane. **b**, The schematic for the interface (armchair). **c,** Numerical results for the projected band structures of configuration (b). **d,** One-half of the surface BZ projected onto the k_{xy} plane, **e**, The schematic for the interface. **f,** Numerical results for the projected band structures of configuration (e). In these two cases, H and H‘ points are projected onto the same point of the surface BZ. The acoustic valley Chern numbers with opposite signs will cancel out; therefore, their surface states are gapped.

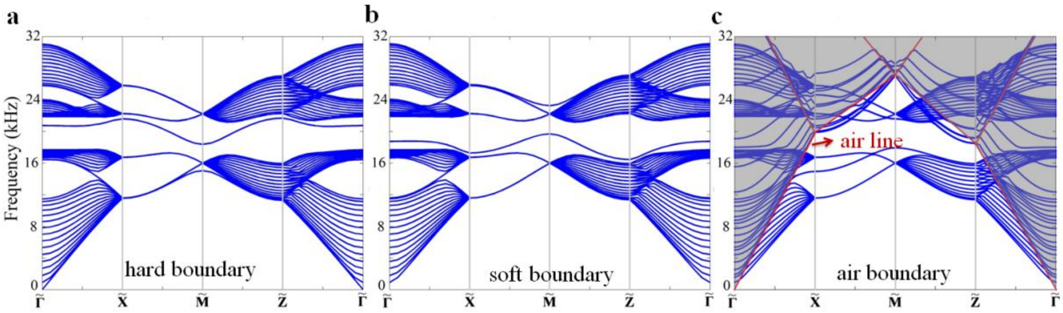

Supplementary Figure 5 | Surface states with three typical boundaries in acoustic system. a, Hard**, b,** Soft and **c,** Air boundaries. In our acoustic system, the acoustic pseudospins are constructed by crystal symmetry. The vacuum or air boundary cannot keep such symmetry. Consequently, the there will open a gap. Red lines in (c) represent the air line of sound. Shadow region indicates where the sound will be scattered into the air.

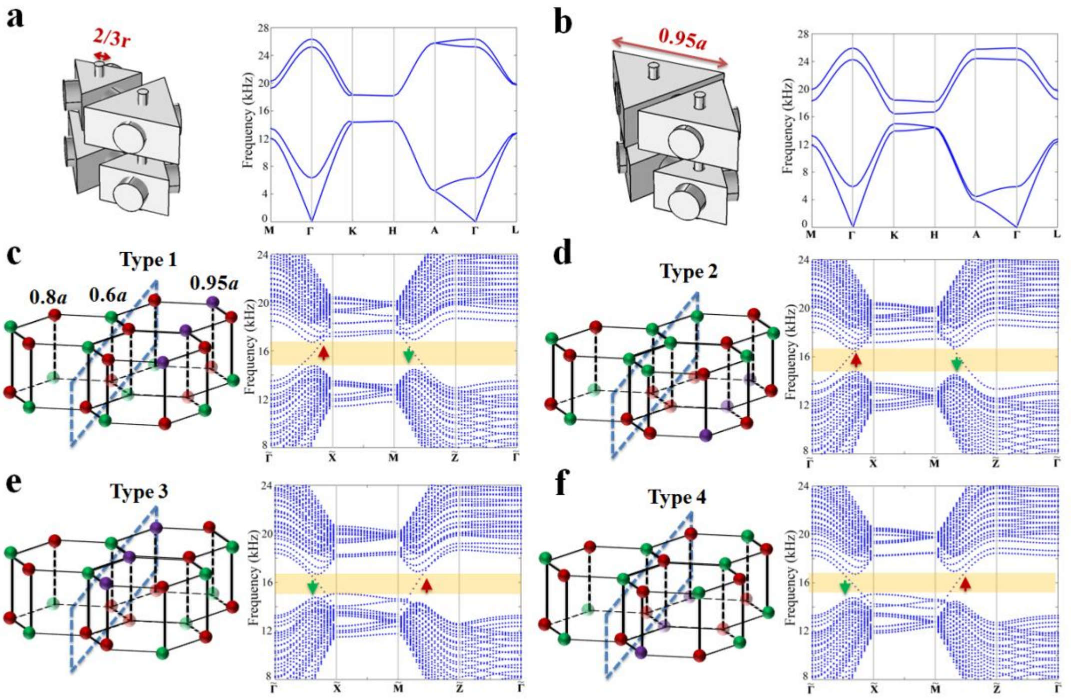

Supplementary Figure 6 | Acoustic topological pseudospin-valley states with a pair of acoustic pseudospins. a, The schematic of unit cell and its bulk bandstructures with $C_v^s = +1$. Here, we reduce the radii of vertical rods to be 2/3*r* to form a complete bandgap, the other parameters are the same as those in Fig. 2c of main text. **b,** $C_v^s = 0$ case (the valley Chern numbers at the H point $C_H = +1/2$ for both lower two bands, while $C_{H'} = -1/2$ at the H' point), by enlarging the upper-left triangular prism's sidelength to be 0.95*a*. **c-f,** Four possible configurations of domain wall with above two acoustic crystals and their corresponding projected band structures. There exists a pair of gapless surface states corresponding to acoustic pseudospins (noted as red and green arrows), indicating the robustness of our acoustic topological pseudospin-valley states.

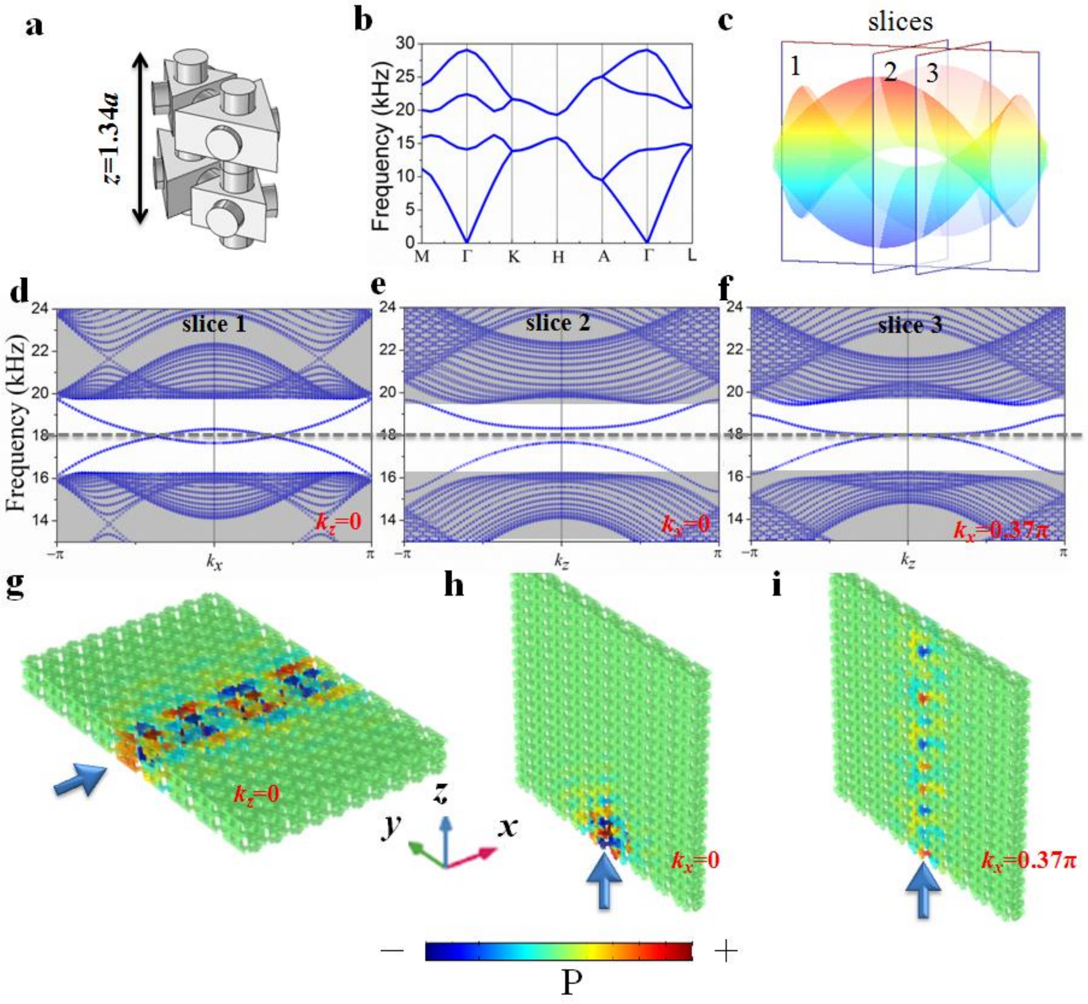

Supplementary Figure 7 | An eye-shape gapless surface states formed by two opposite saddles. a, Unit cell with the height increasing to 1.34*a*, as that in Fig. 4 of main text. **b,** The band structures for bulk states. **c,** The schematic for saddle surface states. Three 2D projected band structure with various *k* slices that are taken from k_z=0, k_x=0 and k_x=0.37π as noted in (c), are shown in **d-f**. The unit of k_x (k_z) is normalized by 1/*a* (1/2*h*). **g-i,** The acoustic pressure field distributions corresponding to (d-f), respectively. Operating frequency is marked as dashed line.

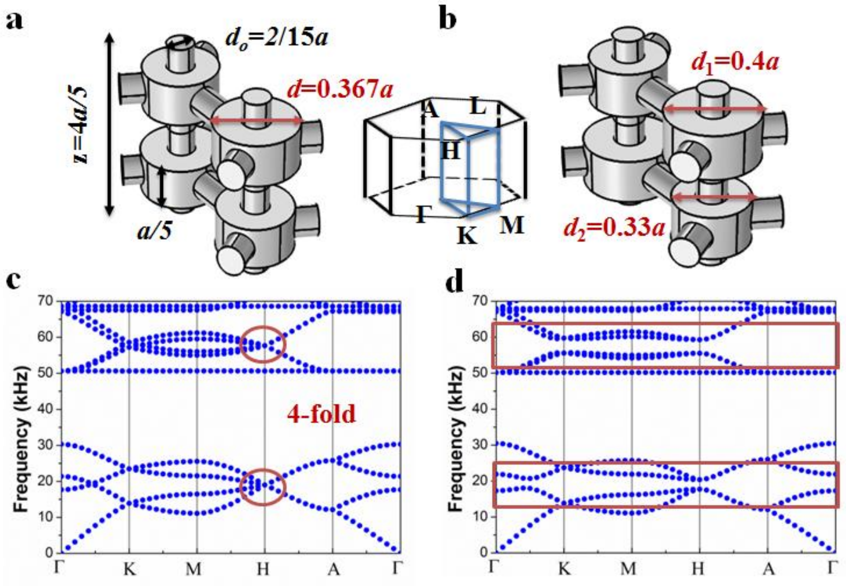

Supplementary Figure 8 | 3D acoustic topological pseudospin-valley states based on cylinder-shape acoustic atoms. In our model, the intrinsic acoustic wave is longitudinal wave, which is non-sensitive to the shape. For example, we can use cylinder-shape acoustic atoms to realize the same topological properties. **a,** Unit cell consists of identical cylinder-shape acoustic atoms. **b,** Unit cell consists of two different cylinder-shape acoustic atoms with glide symmetry. Their band structures are shown in **c** and **d**, respectively. We can find that the four-fold degenerated point can split into two two-fold ones separated by a complete band gap. It should be noticed that not only the first four bands but also higher order bands will act the similar topological behaviors, as indicated in red rectangle boxes.

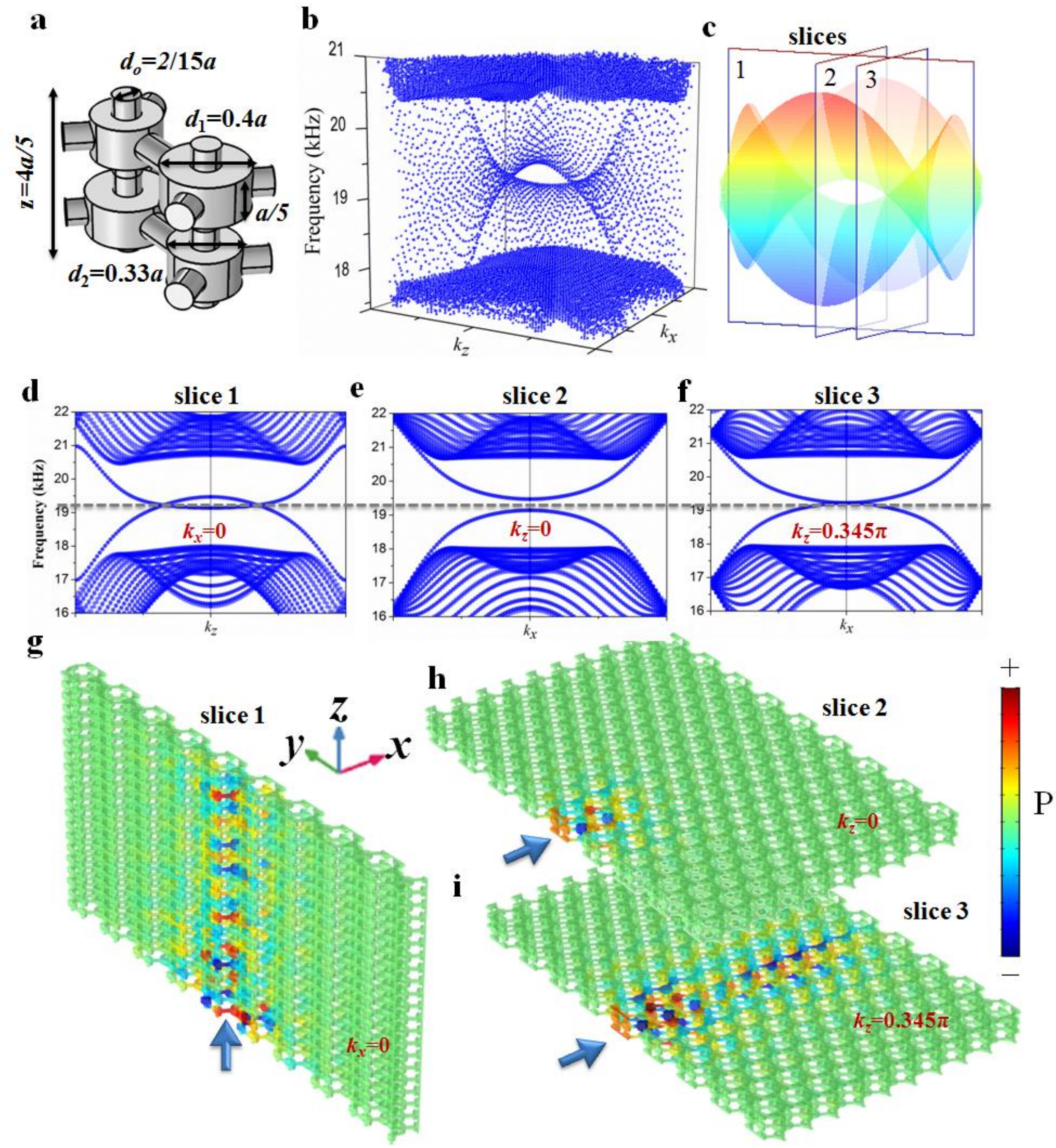

Supplementary Figure 9 | Saddle surface states of cylinder-shape acoustic atoms. a, Unit cell consists of two different cylinder-shape acoustic atoms as Supplementary Figure 8b. **b,** Numerical result and **c,** The Schematic picture for the surface states clearly show that the gapless edge states are constructed by two opposite saddle surfaces. Three 2D projected band structure with various k slices that are taken from k_x=0, k_z=0 and k_z=0.345π noted in (c), are shown in **d-f**. The unit of k_x (k_z) is normalized by 1/a (1/2h)**. g-i,** The acoustic pressure field distributions corresponding to (d-f), respectively. In this case, the gapless saddle surface states open an eye along k_z direction.

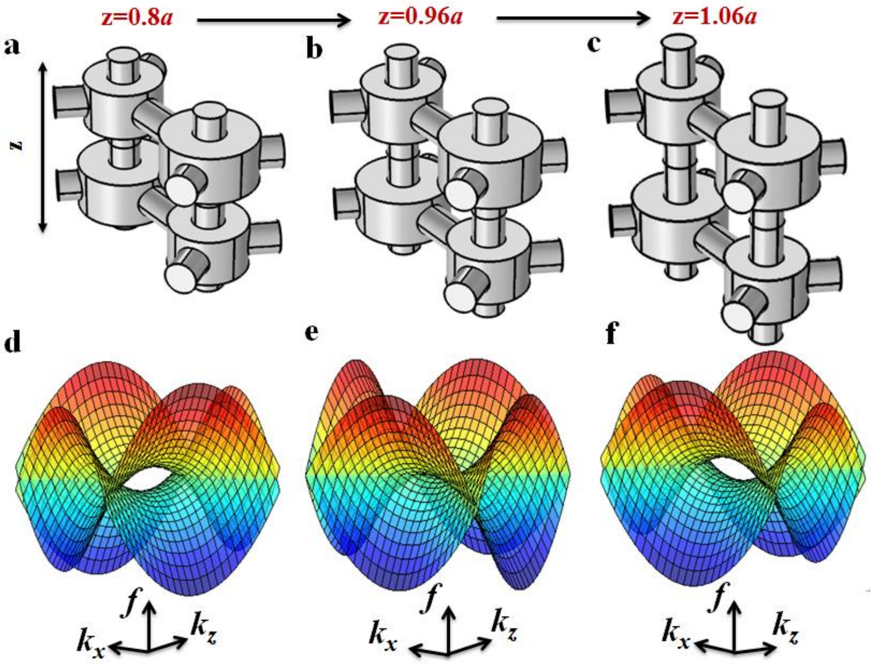

Supplementary Figure 10 | Adjustable saddle surface states. a-c, Unit cells with different heights, 0.8*a*, 0.96*a* and 1.06*a* (other parameters are the same as those in Supplementary Figure 8). **d-f,** Schematics of surface states corresponding to (a-c). With increasing height, the eye formed by two opposite saddle surfaces will open along k_z direction, to close (two saddle points touching each other), then to reopen along k_x direction.

Supplementary Note 1

In this section, we explicitly calculate the topological index, *i.e.*, pseudospin-valley Chern number of our model. As shown in Fig. 1e of main text, there exists a four-fold degeneracy at H (H') point. Near such degenerate point, the system can be described by the following four-band low-energy effective Hamiltonian:

$$H = v_f\left(\chi k_x \sigma_x + k_y \sigma_y\right) - 2t_z cosk_z \tau_x + m\tau_z\sigma_z, \tag{1}$$

where v_f denotes the velocity, t_z represents the interlayer hopping along the z-direction, $m > 0$ is the alternating intralayer inversion breaking mass for each acoustic atom, $\sigma_{i=x,y,z}$ and $\tau_{i=x,y,z}$ are Pauli matrices acting in the intralayer subspace and layer pseudospin subspace, respectively, and $\chi = +1\,(-1)$ for the valley along the K-H (K'-H') lines (see Figs. 1d-f of main text). By diagonalizing Supplementary Equation 1, the energy can be written as:

$$E = \eta\sqrt{\left(2t_z cosk_z \pm v_f k_\perp\right) + m^2}, \tag{2}$$

where $\eta = +1\,(-1)$ for upper (lower) bands. For a nonzero m, a full bulk band gap can be created. As a result, by treating k_z as a parameter, the system can be decoupled into a collection of 2D insulator slices. To characterize the topological property of each 2D slice, we resort to the pseudospin-valley Chern number by projecting the pseudospin operator τ_z onto two lower bands subspace of H as[1]:

$$P_v\tau_z P_v = \begin{pmatrix} \langle\phi_1|\tau_z|\phi_1\rangle & \langle\phi_1|\tau_z|\phi_2\rangle \\ \langle\phi_2|\tau_z|\phi_1\rangle & \langle\phi_2|\tau_z|\phi_2\rangle \end{pmatrix}, \tag{3}$$

where ϕ_1 and ϕ_2 refer to the wavefunctions of the first and the second bands (two lower bands), respectively. Diagonalization of $P_v\tau_z P_v$ leads to two branches (corresponding to a pair of acoustic pseudospins) with eigenvalues $\lambda_\pm$ and associated eigenvectors $\Psi_\pm$. As long as $\lambda_\pm \neq 0$, the pseudospin-spectrum gap always exists, thus enabling one to define the pseudospin-valley Chern number for each branch.

For example, the valley Chern number at H (H') point for a pair of acoustic pseudospins can be described as:

$$\mathrm{C}^{\pm}_{\mathrm{H(H')}} = \frac{1}{2\pi}\int_{\mathrm{H(H')}} d^2k\,\Omega_\pm, \tag{4}$$

where $\Omega_\pm = -2Im\left\langle\frac{\partial\Psi_\pm}{\partial k_x}\middle|\frac{\partial\Psi_\pm}{\partial k_y}\right\rangle$ is the Berry curvature distributed around each valley in the k_{xy} plane.

The valley Chern numbers at H and H' points can be analytically obtained[1]:

$$C_{H}^{\pm} = \pm\frac{sgn(m)}{2} = \pm\frac{1}{2} \; and \; C_{H'}^{\pm} = \mp\frac{sgn(m)}{2} = \mp\frac{1}{2}. \tag{5}$$

The pseudospin-valley Chern number can then be given by[2]:

$$C_{V}^{S} = \frac{(C_{H}^{+} - C_{H'}^{+}) - (C_{H}^{-} - C_{H'}^{-})}{2} = +sgn(m) = +1. \tag{6}$$

Tight-binding calculations

We can also use such graphene-multilayer tight-binding model composed of intrinsic spinless acoustic atoms to figure out the band structures under open-boundary conditions of 40 sites along the y-direction with a zigzag domain-wall configuration introduced at $y = 20$ (Fig. 2b in the main text). The results based on tight-binding model are shown in Supplementary Figure 11, which are consistent with those in the main text obtained from the numerical method (Figs. 2c and 2d in the main text).

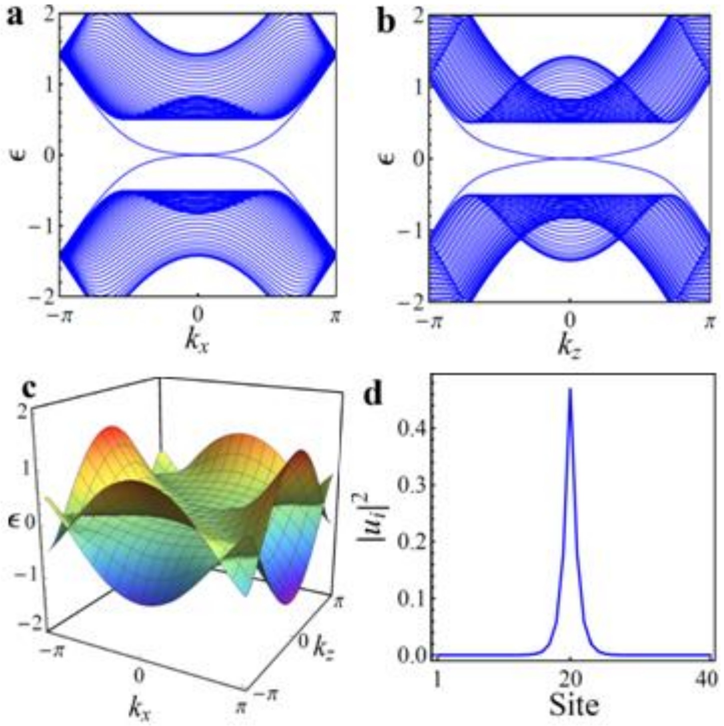

Supplementary Figure 11 | Energy spectrum based on tight-binding method for 40 sites along the y-direction with a domain-wall configuration at the y=20 site. The intralayer nearest hopping t within each layer is chosen as the energy unit, and $m = 0.5t\,(-0.5t)$ for $y \leq 20$ $(y > 20)$. **a,** Energy as a function of k_x in the $k_z = 0$ slice. **b,** Energy as a function of k_z in the $k_x = 0$ slice. **c,** The energy dispersion of the saddle surface state in the k_{xz} plane, which is localized near the domain wall at y=20 lattice site, as verified by the wavefunction distribution of the lower band of the saddle surface states along the y-direction, shown in **d.**

Supplementary References:

1. D. N. Sheng, Z. Y. Weng, L. Sheng, and F. D. M. Haldane, Quantum Spin-Hall Effect and Topologically Invariant Chern Numbers, Phys. Rev. Lett. **97**, 036808 (2006).

2. Ezawa, M. Topological Kirchhoff law and bulk-edge correspondence for valley Chern and spin-valley Chern numbers. *Phys. Rev. B* **88**, 161406 (2013).
